# Epidemiology of Soft Tissue Sarcoma in Iran: Four‐Year National Cancer Registry Data Report (2014–2017)

**DOI:** 10.1002/cnr2.70118

**Published:** 2025-01-10

**Authors:** Mohammad Sajed Dehghan Banadaki, Vahid Rahmanian, Saeed Hosseini, Seyyed Mohammad Hossein Hosseini, Narjes Hazar

**Affiliations:** ^1^ MD, School of Medicine Shahid Sadoughi University of Medical Sciences Yazd Iran; ^2^ Assistant Professor in Epidemiology, Department of Public Health Torbat Jam Faculty of Medical Sciences Torbat Jam Iran; ^3^ Ph.D. Candidate in Epidemiology, Center for Healthcare Data Modeling, Department of Biostatistics and Epidemiology, School of Public Health Shahid Sadoughi University of Medical Sciences Yazd Iran; ^4^ Department of Epidemiology, School of Public Health Iran University of Medical Sciences Tehran Iran; ^5^ MSC in Epidemiology, Department of Biostatistics and Epidemiology Shahid Sadoughi University of Medical Sciences Yazd Iran; ^6^ MD, Assistant Professor in Community Medicine, Diabetes Research Center Shahid Sadoughi University of Medical Sciences Yazd Iran

**Keywords:** epidemiology, incidence rate, Iran, soft tissue sarcoma

## Abstract

**Introduction:**

An uncommon and diverse class of cancers originating from mesenchymal tissues is designated as soft tissue sarcoma (STS). To develop effective preventive and treatment strategies for STS, it is essential to gain a deeper understanding of the epidemiological trends associated with the disease. This research will analyze the 4‐year age‐standardized incidence rate (ASIR) and geographical distribution of STS in Iran in great detail.

**Methods:**

The study population comprised 4968 cases of STS recorded in the Cancer Registry System between 2014 and 2017. The demographic data examined included gender, place of residence, and year of diagnosis. The age‐standardized rate (ASR) of STS incidence was calculated for each location using the World Standard Population. The data were examined using the program ArcMap10.5. The geographic distribution of STS was investigated using the Moran test.

**Results:**

The ASRs for STS in Iran from 2014 to 2017 were recorded as 1.25 (ASR in male: 1.47, ASR in female: 1.06), 1.36 (ASR in male: 1.46, ASR in female: 1.29), 1.37 (ASR in male: 1.52, ASR in female: 1.21), and 1.78 (ASR in male: 1.58, ASR in female: 1.98), respectively. In 2014 and 2015, age‐standardized incidence at the national level showed a statistically significant regional dispersion that appeared as a clustering pattern, according to Moran's test. However, in 2016 and 2017, this dispersion failed to become statistically significant. Interestingly, men had a greater rate of STS incidence than females. As age grows, ASIR shows a steadily rising trend. The most important gains are shown in the 55–59 age group, which peaked at 4.535 in 2017, and the 80–84 age group, which peaked at 10.848 in the same year.

**Conclusion:**

The incidence of STS in Iran is lower than the global average. The discrepancies in gender disparities, regional distribution, and incidence rates underscore the complexity of STSs. The findings of this study may assist healthcare professionals and policymakers in the development of region‐specific plans for the treatment, early detection, and prevention of STSs.

**Abbreviations**
ASIR: age‐standardized incidence rate
ASR: age‐standardized rate
BS: bone sarcomas
CR: crude incidence rate
CT: computed tomography
GIS: geographic information system
ICD‐10: international statistical classification of diseases and related health problems 10th revision
INPCR: Iranian National Cancer Registry
STS: soft tissue sarcomas

## 1 Introduction

Sarcomas are malignant tumors derived from mesenchymal tissue, which are broadly categorized into those originating in the bone and those originating in soft tissue. Bone sarcomas (BS) encompass osteosarcomas, chondrosarcomas, and Ewing sarcomas, whereas soft tissue sarcomas (STS) constitute a heterogeneous group of malignancies originating from mesenchymal tissue throughout the body [1]. Although uncommon, STS are a varied category with a wide range of histological characteristics [2]. Retroperitoneal and primarily intraperitoneal affect the lower limbs and the pelvic girdle [3]. STS may spread to other body parts, showing signs of local invasiveness and destructive development patterns. According to the American Cancer Society, they represent 15% of pediatric cancers and 1% of adult malignancies, respectively, with over 9000 new cases being identified in the United States each year [4]. Their histology is very variable.

Most STS affect people over 60, and over 50% of cases result in death. They are also linked to a high mortality rate [5]. The most recent classification from the World Health Organization recognizes over 50 subtypes of STS. STS are distinguished by a variety of subtypes, each exhibiting distinctive clinical and pathological characteristics. Notable among these subtypes are fibrous histiocytomas, liposarcomas, rhabdomyosarcomas, and synovial sarcomas. Liposarcomas are the most frequently occurring malignant STS, while fibrous histiocytomas and muscular sarcomas are more prevalent in males. Conversely, synovial sarcomas are more prevalent in females [1, 2].

The most common presentation is a painless lump that gradually increases in size. Tumors located in the head, neck, and distal limb are frequently smaller at the time of diagnosis due to the fact that they are identified at an earlier stage. Tumors in other locations are typically larger. Nevertheless, retroperitoneal and thigh tumors may exhibit considerable growth prior to detection. Spherically expanding STS may sporadically invade nearby structures. Individuals may present with distal limb edema, paraesthesia, or symptoms associated with bladder involvement, which may be indicative of the local pressure impact [3].

Despite the continued rarity and complexity of STS, our comprehension of their epidemiological patterns is gradually expanding. The incidence of sarcomas in the United States increased from 6.8 cases per 100 000 individuals in 2002–7.7 cases per 100 000 individuals in 2014. In 2018, STS constituted approximately 0.8% of all cancer cases in the United States, with an estimated 13 000–16 000 new cases and 5000–6000 fatalities annually [4]. STS represent approximately one in two instances of cancer globally, comprising 0.6% of all cancer cases and 0.7% of all cancer‐related mortalities. Malignant bone tumors account for just over 10% of all sarcomas; conversely, STS represent the majority of diagnosed cases. The rarity of the disease and the wide range of its subtypes may present challenges to epidemiological research on sarcomas. A further study, which examined the incidence of sarcomas in Shanghai between 2002 and 2015, revealed an age‐standardized incidence rate (ASIR) of 3.4 per 100 000 people [5].

The crude incidence rate in the general population of Iran is approximately 3.2 cases per 100 000 individuals. There was an increase in incidence with age for both STS and BS, with rates of 2.7 and 0.5 per 100 000 individuals, respectively. In 16.47% and 83.53% of cases, there was a male predominance among patients with BS and STS. The ASIR was calculated to be 2.8 and 2.6 for STS and 0.51 and 0.37 for BS, respectively, for both men and women [6].

In individuals under the age of 16, the incidence ratio of BS to STSs is one to two; in adults, it is three to sixteen. In 2012, 11 280 new cases of STSs were recorded in Iran, with 6110 occurring in males and 5170 in females [7]. The three most prevalent forms of paediatric sarcoma in Iran are PNET, rhabdomyosarcoma and osteosarcoma [8]. The most frequently occurring STSs in adults are malignant fibrous histiocytoma and synovial sarcoma, whereas osteosarcoma represents the most prevalent BS [6].

The incidence of sarcoma varies between different countries. This intricate scenario may be attributed to a multitude of environmental, behavioral, socioeconomic, genetic, and cultural variables [9, 10].

The rarity of STSs and the broad spectrum of histological subtypes present unique challenges to epidemiological research. The diversity in diagnostic criteria and reporting practices across healthcare systems represents an additional challenge in the collection and interpretation of epidemiological data. These factors contribute to the difficulty in accurately assessing the true incidence, prevalence, and distribution patterns of STSs on a global scale. It is imperative that diagnostic protocols are improved and standardized if these challenges are to be overcome and our understanding of sarcoma epidemiology is enhanced. The objective of this study is to contribute to the aforementioned effort by utilizing data from the Iranian National Cancer Registry to explore the regional distribution and age‐standardized rates of STSs in Iran from 2014 to 2017.

## 2 Methods

### 2.1 Study Design

This study employs a descriptive methodology to examine the data from the National Cancer Registry database. The study encompasses all patients with STS documented in the Cancer Registry System between 2014 and 2017. The variables to be analyzed include age, gender, and province of residence, diagnostic technique, and morphology. Subsequently, incidence rates are published by the World Health Organization in accordance with the global standard population. A comprehensive review of data from all regions of the country was conducted, with particular attention paid to the total number of cases and the average incidence rates observed in each province. A prior paper provided specifics on the data‐gathering techniques used by the Iranian National Population‐based Cancer Registry [11].

### 2.2 Definition

It should be noted that pathology reports are not the sole source of information utilized within the present framework of the population‐based cancer registry system for the diagnosis of cancer. The case was classified as positive upon the discovery of corroborative paraclinical evidence within the patient's medical records, which were subsequently verified by a qualified medical professional, or the presence of a positive pathology report indicating the presence of sarcoma. Moreover, the patient's death certificate was duly recorded if it is mentioned that the patient had STS. Consequently, the case files of all patients with supporting documentation, including CT scan results indicating the presence of STS, were duly recorded. Moreover, the diagnostic process was meticulously documented to facilitate future evaluations. This enabled the differentiation between STS diagnosed via pathology and cytology, those identified through paraclinical and clinical reports, and those diagnosed using data from death certificates. It should be noted that STS was classified according to the International Classification of Diseases for Oncology (ICD‐O) using code C49.

## 3 Crude Incidence, ASR, and ASIR

A crude incidence rate is a measure of the number of new cases of a disease (in this case, sarcoma) that occur in a population over a specified period of time. Such figures are frequently expressed as a rate per 100 000 inhabitants. To calculate the crude rate of sarcoma in each province, the number of new cases of STS was divided by the total population, and the result was then multiplied by 100 000. In this research, the ASR was calculated in accordance with the techniques established by the World Health Organization. Prior to calculating the ASR, the crude incidence rate (CR) was determined for each province. Subsequently, the Global Health Organization's global standard population was employed to normalize the aforementioned rate. The following formula may be used to determine ASR:

$$\mathrm{ASR}=\frac{\sum \left(\mathrm{CRi}\times \mathrm{Pi}\right)}{\sum \mathrm{Pi}}\times \mathrm{WSR}$$

- ASR represents the age‐standardized rate.
- CRi denotes the crude incidence rate for each province
- Pi is the population of the province
- WSR is the world standard rate

Another crucial statistic in epidemiological research is the ASIR. In contrast to the ASR, which is calculated using the global standard population, the ASIR is derived using an age‐group‐specific standard population. The ASIR and ASR are derived through a similar mathematical procedure; however, the former utilizes a standard population specific to a particular age range, as opposed to the global standard population employed in the latter.

## 4 Statistical Analysis

The initial step involved the retrieval of the number of STS for each province from the national STS dataset, which was available in an Excel file. This was done to compute the crude and age‐specific incidence rates. Subsequently, the age‐ and crude‐specific incidence rates were calculated using the population data for each province and classified according to the province of residence. Moreover, the ASR for each province were calculated using the global standard population. These data were further examined using ArcMap 10.5 software and other geographic information system tools. The geographic distribution and clustering of STS were examined using Moran's index tests. The Moran's Index represents an effective method for determining the geographical proximity of comparable data points. A positive value indicates the presence of a clustering pattern, whereby data points of a similar nature are situated in closer proximity to one another than would be expected under the given circumstances. Conversely, a negative score indicates that the data points are frequently situated at greater distances than would be expected. The Moran's Index is of great importance in the revelation of geographical connections and trends present within a given dataset.

## 5 Results

**FIGURE 1:**
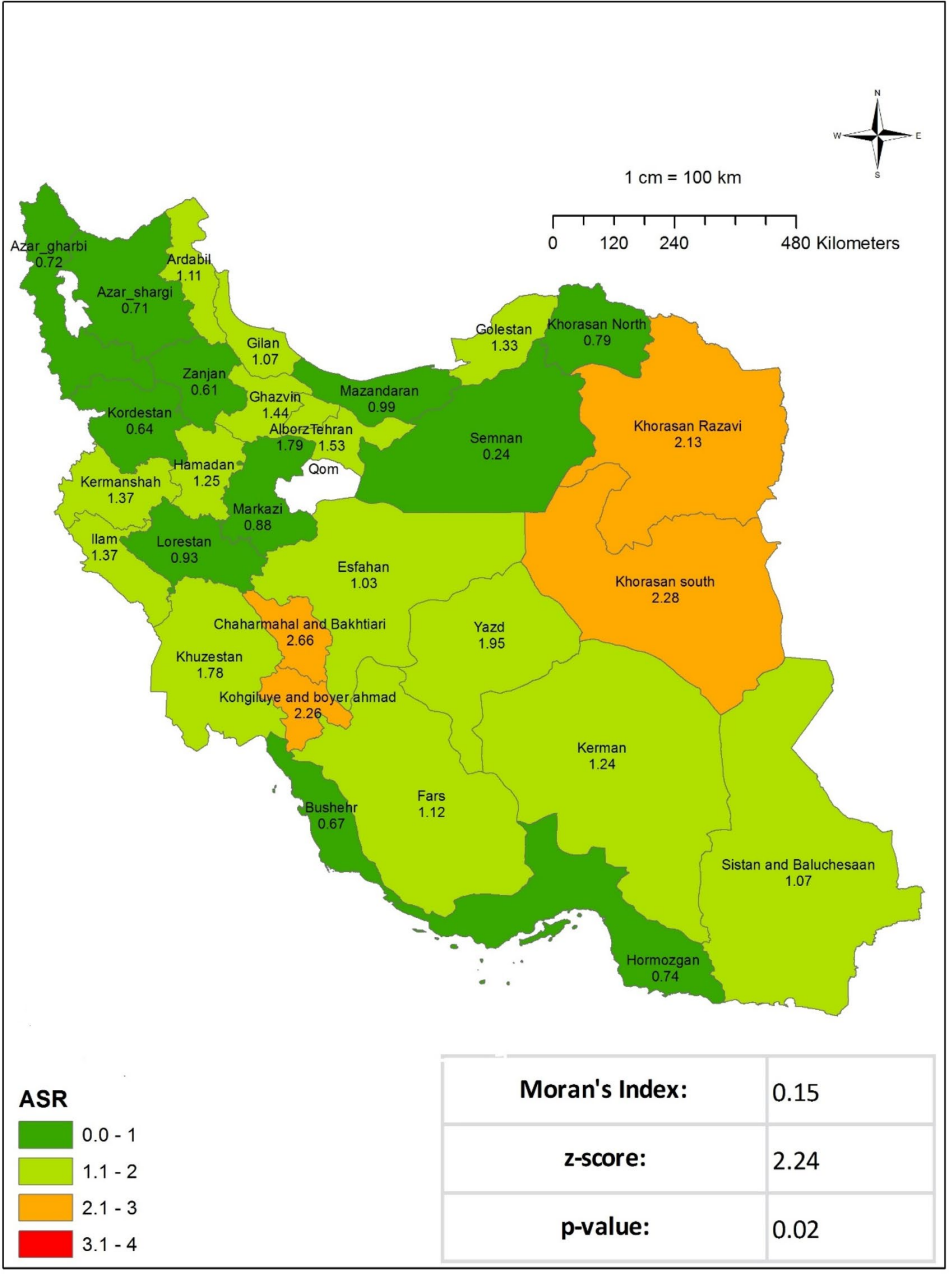
Age‐standardized incidence rate (ASIR) of soft tissue sarcoma per 100 000 populations in Iran in 2014.

**FIGURE 2:**
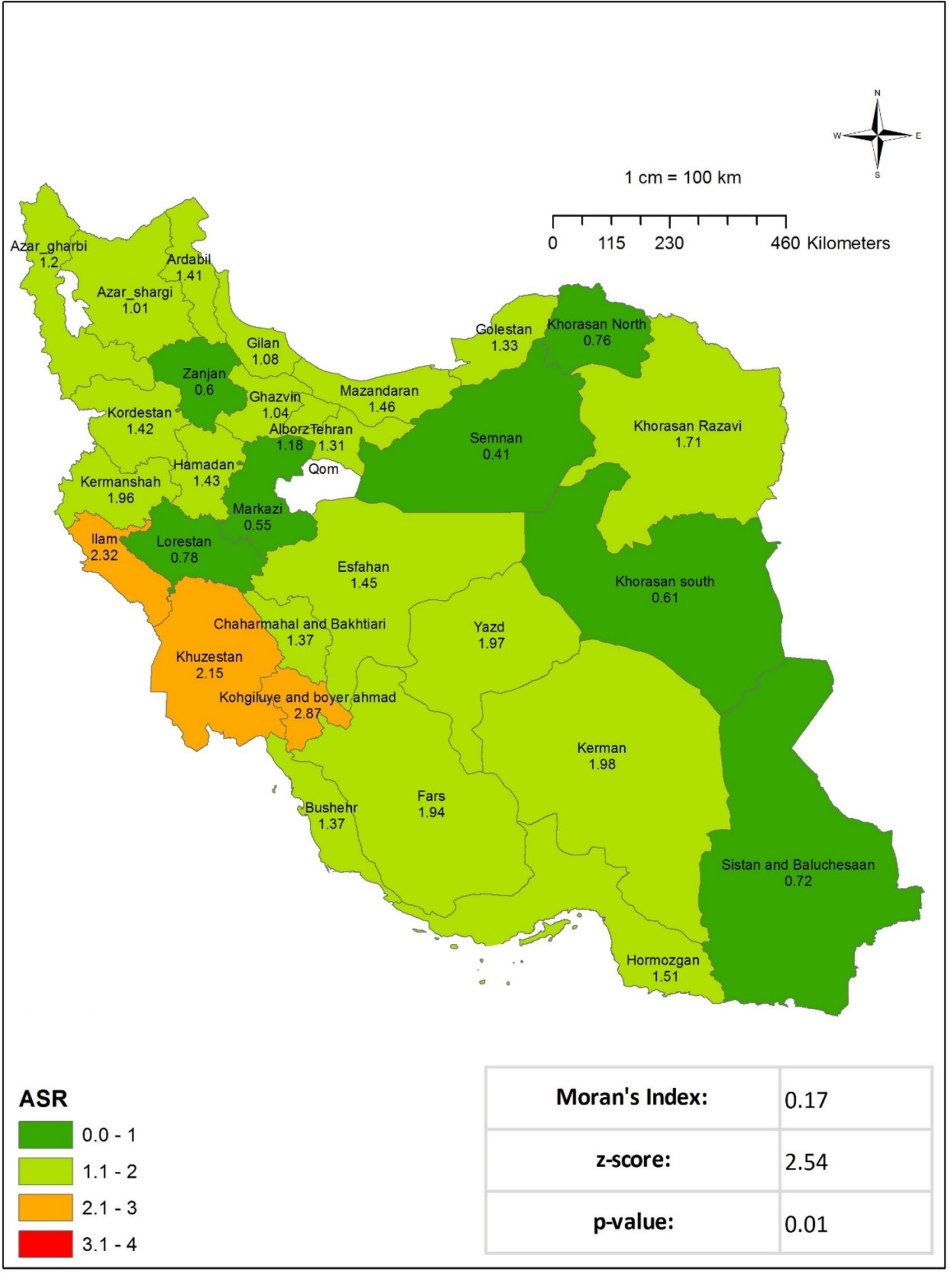
Age‐standardized incidence rate (ASIR) of soft tissue sarcoma per 100 000 populations in Iran in 2015.

**FIGURE 3:**
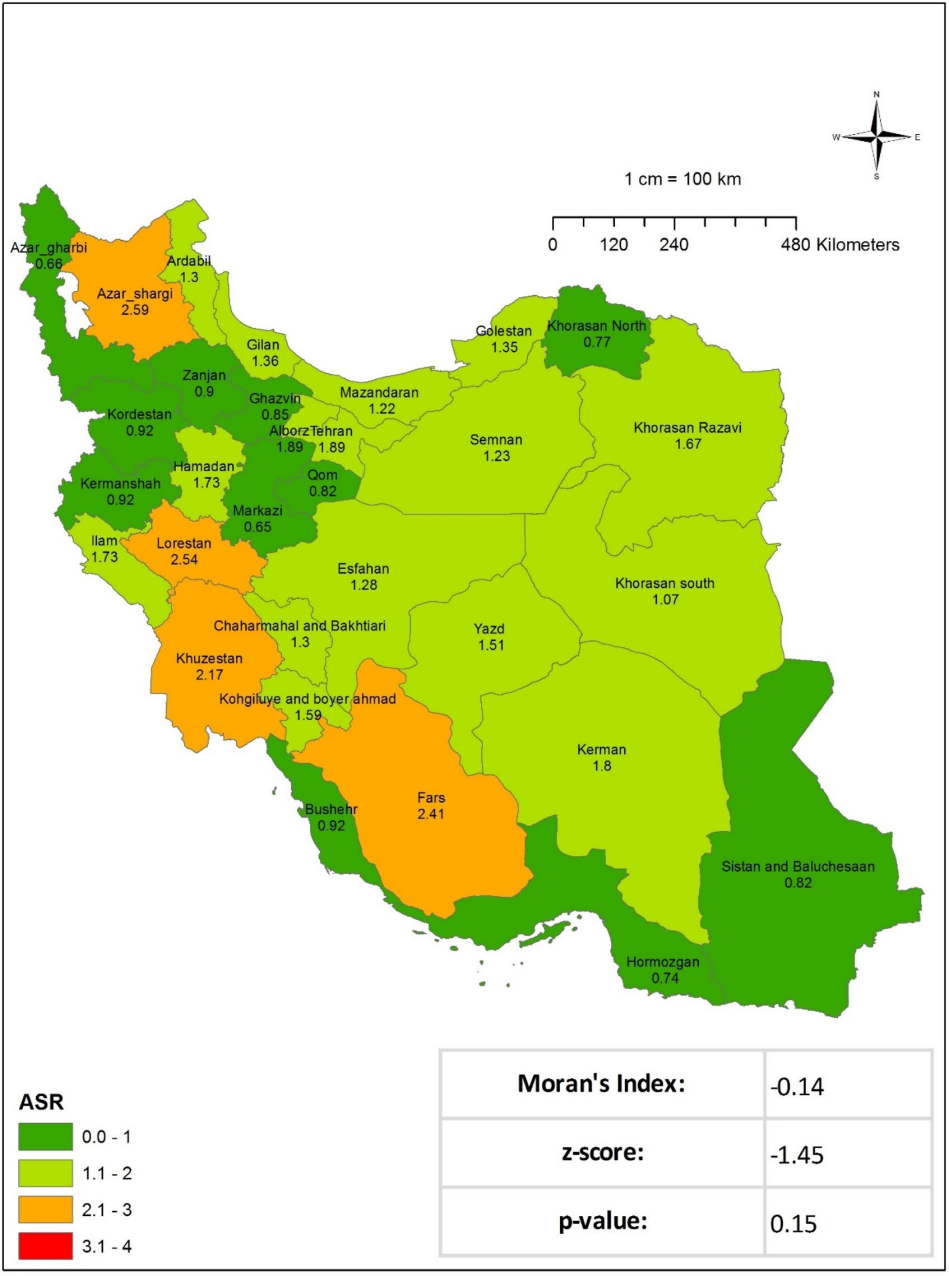
Age‐standardized incidence rate (ASIR) of soft tissue sarcoma per 100 000 Iran population in 2016.

**FIGURE 4:**
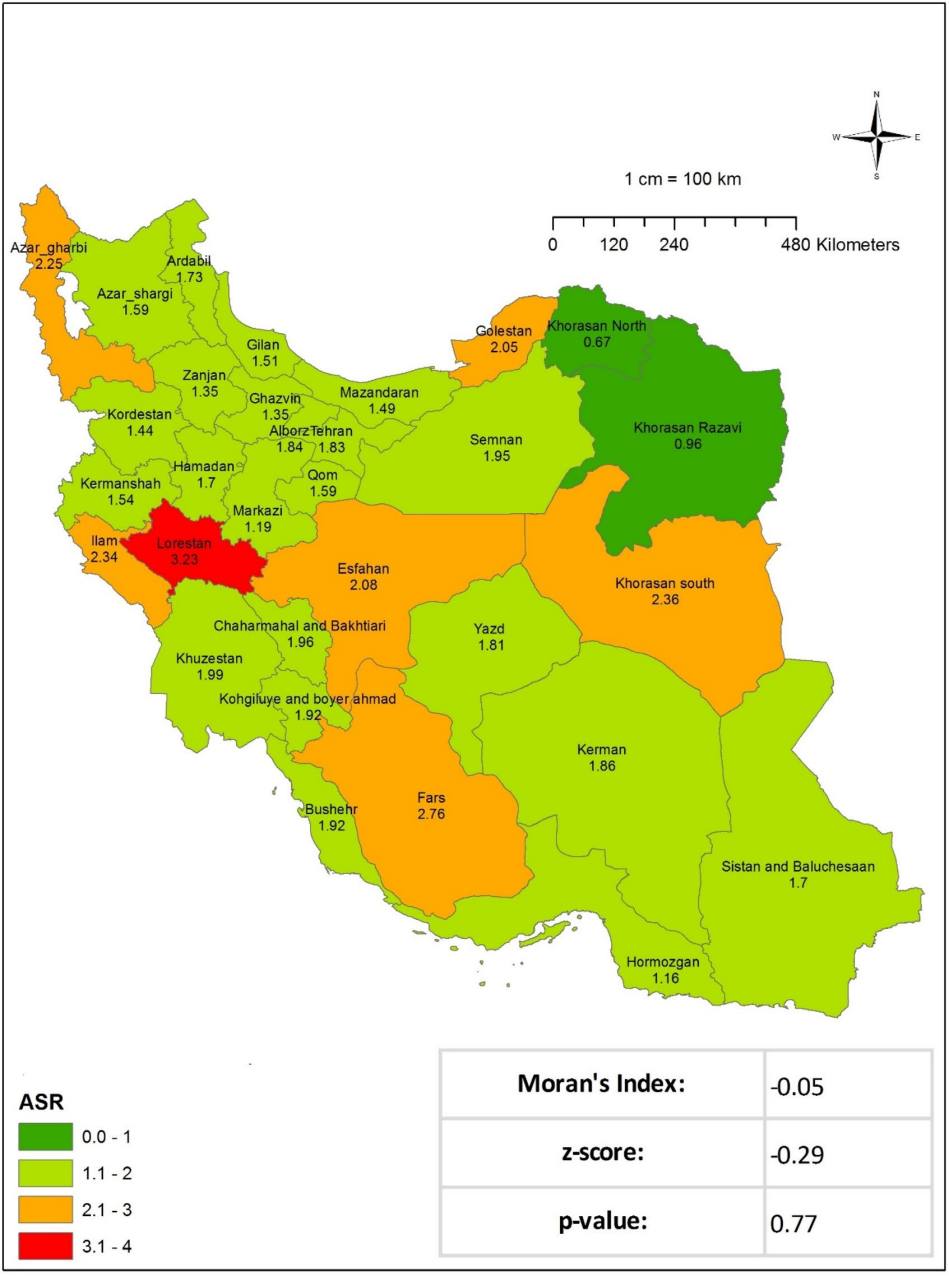
Age‐standardized incidence rate (ASIR) of soft tissue sarcoma per 100 000 populations in Iran in 2017.

This study undertook a review of 4968 cases of STS registered in the Cancer Registry System between the years 2014 and 2017. Of the total number of cases, 1010 patients received their initial diagnosis in 2014. In 2014, the ASIR for STS in the nation was 1.25, corresponding to a crude incidence rate of 1.2. The areas with the highest ASIR were South Khorasan (2.28), Kohgiluyeh and Boyer‐Ahmad (2.25), and Chaharmahal and Bakhtiari Province (2.66). At the national level, the results of Moran's test indicated a notable geographical dispersion of this occurrence, indicating a cluster‐type dispersion (Moran's Index: 0.15, *p* value: 0.02) (Figure 1 and Table 1). A review of the registry data revealed that there were 1149 cases of STS recorded nationally in 2015. The ASIR was 36.1, and the crude incidence rate for the entire nation was 36.1. The regions exhibiting the most elevated ASIR were Khuzestan (2.15), Isfahan (2.31), Kohgiluyeh, and Boyer‐Ahmad Province (2.87). The results of Moran's test indicated the presence of a cluster type (Moran's Index: 0.17, *p* value: 0.01) and a statistically significant geographical dispersion (**Figure** 2 and Table 1). In 2016, there were 1317 instances of STS registered nationally. With an ASIR of 1.37, the crude incidence rate for the entire country was 1.45. The three regions with the highest ASIR were Fars (2.41), Lorestan (2.54), and Alborz Province (2.58). As illustrated in Figure 3 and Table 1, the results of Moran's test indicate that there is no statistically significant evidence of regional dispersion for this occurrence across the country (Moran's Index: −0.14, *p* value: 0.15). In 2017, 1492 documented cases of STS were registered at the national level. With an ASIR of 1.78, the crude incidence rate for the entire country was 1.79. As given in Table 1, the regions exhibiting the highest ASIR were South Khorasan (2.35), Fars (2.75), and Lorestan Province (3.22). Moran's test revealed no statistically significant regional variation in the prevalence of this occurrence throughout the nation (Moran's Index: −0.05, *p* value: 0.77; see **Figure** 4 and Table 1). In addition, the research examined each documented case of sarcoma in the Cancer Registry System among male patients in the following years: 2014 (577 cases), 2015 (631 cases), 2016 (735 cases), and 2017 (833 cases) Figures 2, 3, 4. The crude incidence rates for sarcoma in men exhibited considerable variation, ranging from 1.37 to 1.95. In contrast, the ASR exhibited a narrower range, falling between 1.47 and 1.98. While the most notable ASIR exhibited fluctuations from year to year, South Khorasan, Chaharmahal and Bakhtiari, Kohgiluyeh and Boyer‐Ahmad, Fars, and Lorestan were consistently among the regions with the highest rates (Table 1 and Figures S1–S4). Similarly, the research examined all instances of female sarcoma reported in 2014 (433 cases), 2015 (518 cases), 2016 (581 cases), and 2017 (659 cases). The crude incidence rates for sarcoma in women exhibited considerable variation, ranging from 1.03 to 1.61. In contrast, the ASIR demonstrated a narrower range, spanning from 1.06 to 1.58. The regions with the most notable ASIR were Chaharmahal and Bakhtiari, Yazd, Kohgiluyeh and Boyer‐Ahmad, Ilam, and Fars, as given in Table 1 and in the Figures (S5–S8).

**TABLE 1:** Number of sarcoma cases, crude incidence rate, total standardized incidence rate (ASR), and standardized incidence rate (ASR) for women and men by province of residence during the years 2014–2017 in Iran.

Residence	2014					2015					2016					2017				
	*N*	Incidence	ASR in male	ASR in female	ASR	*N*	Incidence	ASR in male	ASR in female	ASR	*N*	Incidence	ASR in male	ASR in female	ASR	*N*	Incidence	ASR in male	ASR in female	ASR
Iran	1010	1.20	1.47	1.06	1.25	1149	1.36	1.46	1.29	1.36	1317	1.45	1.52	1.21	1.37	1492	1.79	1.98	1.58	1.78
East Azerbaijan	71	1.86	2.12	1.45	1.79	49	1.28	1.59	0.79	1.18	74	1.89	1.86	1.87	1.89	77	1.95	1.76	1.93	1.84
West Azerbaijan	34	1.06	1.13	1.07	1.11	47	1.45	1.19	1.63	1.41	43	1.32	1.86	0.75	1.30	55	1.67	1.39	2.08	1.73
Ardabil	11	0.86	1.16	0.92	1.03	18	1.40	0.84	2.03	1.45	19	1.50	1.65	0.91	1.28	28	2.20	2.45	1.67	2.08
Isfahan	70	1.40	1.54	1.22	1.37	123	2.44	2.66	1.99	2.32	96	1.87	1.80	1.66	1.73	136	2.63	2.28	2.39	2.34
Alborz	17	0.67	0.83	0.60	0.71	26	1.00	1.39	0.61	1.01	65	2.40	2.83	2.35	2.59	48	1.73	1.68	1.48	1.59
Ilam	5	0.87	0.25	1.09	0.72	6	1.03	1.10	1.35	1.20	4	0.69	0.75	0.55	0.66	13	2.22	1.74	2.92	2.25
Bushehr	8	0.73	0.80	0.55	0.67	12	1.07	1.11	1.57	1.37	9	0.77	1.66	0.13	0.92	19	1.60	2.61	1.17	1.92
Tehran	194	1.54	1.81	1.25	1.53	172	1.36	1.40	1.24	1.31	275	2.07	2.10	1.69	1.89	274	2.03	2.09	1.57	1.83
Chaharmahal and Bakhtiari	22	2.38	3.33	2.08	2.66	17	1.82	2.61	1.17	1.37	13	1.37	1.78	0.79	1.30	20	2.09	0.53	3.40	1.96
South Khorasan	16	2.11	3.98	0.68	2.28	6	0.78	0.52	0.68	0.61	8	1.04	1.36	0.77	1.07	18	2.28	3.46	1.28	2.36
Razavi Khorasan	122	1.95	2.50	1.80	2.13	103	1.62	1.78	1.67	1.71	104	1.62	1.83	1.48	1.67	60	0.92	1.30	0.63	0.96
North Khorasan	7	0.78	0.76	0.84	0.79	6	0.66	0.43	1.06	0.76	7	0.81	0.40	1.14	0.77	6	0.70	0.77	0.56	0.67
Khuzestan	73	1.55	1.90	1.66	1.78	93	1.94	2.81	1.51	2.15	94	2.00	2.31	2.06	2.17	90	1.90	2.75	1.25	1.99
Zanjan	7	0.67	1.06	0.20	0.61	7	0.66	0.28	0.94	0.60	9	0.85	0.87	1.00	0.90	15	1.41	1.84	0.83	1.35
Semnan	2	0.30	0.20	0.29	0.24	4	0.59	0.46	0.37	0.41	9	1.28	0.77	1.69	1.23	13	1.81	1.98	1.89	1.95
Sistan and Baluchestan	24	0.88	1.35	1.22	1.07	17	0.61	0.82	0.61	0.72	15	0.54	0.64	0.99	0.82	32	1.13	2.29	1.12	1.70
Fars	54	1.14	1.21	1.04	1.12	92	1.92	2.12	1.77	1.94	115	2.37	3.35	1.48	2.41	134	2.73	2.80	2.71	2.76
Qazvin	17	1.37	1.52	1.40	1.44	13	1.04	1.36	0.71	1.04	13	1.02	1.04	0.66	0.85	20	1.55	1.25	1.44	1.35
Qom	—	—	—	—	—	—	—	—	—	—	11	0.85	0.40	1.26	0.82	20	1.51	2.23	0.98	1.59
Kurdistan	10	0.66	0.82	0.46	0.64	22	1.43	1.44	1.39	1.42	15	0.94	1.21	0.65	0.92	26	1.60	1.93	0.94	1.44
Kerman	34	1.11	1.64	0.87	1.24	59	1.89	2.44	1.54	1.98	57	1.80	1.44	2.17	1.80	60	1.87	1.75	1.95	1.86
Kermanshah	30	1.53	1.52	1.24	1.37	39	1.97	2.61	1.35	1.96	22	1.13	1.06	0.80	0.92	33	1.69	2.03	1.10	1.54
Yasuj	12	1.73	2.77	1.61	2.26	18	2.56	2.84	2.72	2.87	11	3.13	2.35	0.71	1.59	14	1.93	3.01	0.70	1.92
Golestan	22	1.18	1.52	1.17	1.33	24	1.26	1.53	1.12	1.33	28	1.50	1.20	1.53	1.35	39	2.07	1.86	2.27	2.05
Gilan	29	1.15	1.05	1.07	1.07	32	1.26	1.23	0.94	1.08	37	1.46	2.09	0.63	1.36	48	1.89	1.84	1.18	1.51
Lorestan	15	0.83	1.38	0.47	0.93	14	0.77	0.73	0.80	0.78	44	2.50	2.97	2.07	2.54	52	2.95	4.12	2.43	3.23
Mazandaran	36	1.14	1.24	0.74	0.99	50	1.57	1.98	0.94	1.46	46	1.40	1.07	1.37	1.22	57	1.71	1.49	1.50	1.49
Markazi	14	0.96	0.93	0.80	0.88	8	0.54	0.59	0.55	0.55	12	0.84	1.05	0.27	0.65	16	1.12	1.84	0.57	1.19
Hormozgan	11	0.66	0.37	1.09	0.74	23	1.35	1.13	1.85	1.51	12	0.68	0.65	0.80	0.74	19	1.05	1.16	1.13	1.16
Hamadan	25	1.40	1.61	0.94	1.25	26	1.45	1.07	1.74	1.43	33	1.90	1.60	1.86	1.73	30	1.73	1.79	1.64	1.70
Yazd	18	1.69	1.82	2.15	1.95	23	2.11	1.82	2.12	1.97	17	1.49	1.46	1.60	1.51	20	1.74	1.39	2.29	1.81

Based on the data analysis from the National Cancer Registry of Iran, between 2014 and 2017, 4968 cases of STS were registered. Among these, 3136 cases (63%) were classified into 10 distinct histopathological subtypes, with the corresponding crude incidence rates (CIR) and ASIR calculated for each year (Table 2). Malignant fibrous histiocytoma showed an increasing trend in CIR from 0.080 in 2014–0.106 in 2017, with ASR peaking at 0.125 in 2015. Myxoid liposarcoma's CIR ranged from 0.055 to 0.071, with relatively stable ASR values between 0.052 and 0.066. Synovial sarcoma, NOS, steadily increased CIR and ASR, reaching 0.077 and 0.071, respectively, by 2017. Dermatofibrosarcoma, NOS, demonstrated a significant rise in CIR from 0.025 in 2014–0.181 in 2017, with ASR increasing from 0.022 to 0.163. Desmoplastic small round cell tumors maintained a relatively stable incidence, with CIR around 0.094 and ASR close to 0.092 by 2017. Undifferentiated pleomorphic sarcoma showed an initial CIR of 0.060 in 2014, peaking at 0.115 in 2016, with ASR following a similar pattern. Leiomyosarcoma, NOS, consistently increased CIR and ASR, reaching 0.105 and 0.103, respectively in 2017. Liposarcoma's CIR rose from 0.029 to 0.060, with ASR increasing from 0.029 to 0.058 over the study period. Sarcoma‐NOS had a high CIR of 0.287 in 2014, with a notable decrease to 0.146 in 2016 before rising again to 0.283 in 2017, and ASR followed a similar trend. Other specified sarcomas CIR ranged from 0.224 to 0.186, with corresponding ASR values fluctuating accordingly (Table 2).

**TABLE 2:** Incidence rates of the ten most common soft tissue sarcoma subtypes in Iran (2014–2017).

Subtype	2014		2015		2016		2017	
	CIR	ASR	CIR	ASR	CIR	ASR	CIR	ASR
Malignant fibrous histiocytoma	0.080	0.087	0.116	0.125	0.102	0.104	0.106	0.103
Myxoid liposarcoma	0.055	0.054	0.067	0.063	0.061	0.052	0.071	0.066
Synovial sarcoma, NOS	0.047	0.043	0.066	0.060	0.076	0.072	0.077	0.071
Dermatofibrosarcoma, NOS	0.025	0.022	0.138	0.113	0.179	0.160	0.181	0.163
Desmoplastic small round cell tumor	0.096	0.096	0.079	0.085	0.087	0.090	0.094	0.092
Undifferentiated pleomorphic sarcoma	0.060	0.068	0.069	0.071	0.115	0.110	0.091	0.094
Leiomyosarcoma	0.039	0.040	0.054	0.058	0.074	0.072	0.105	0.103
Liposarcoma	0.029	0.029	0.038	0.039	0.048	0.046	0.060	0.058
Sarcoma, NOS	0.287	0.299	0.275	0.284	0.146	0.145	0.283	0.290
Other specified sarcomas	0.224	0.233	0.134	0.133	0.206	0.210	0.186	0.174

*Note:* Total: 3136 out of 4968 cases (63%).

Abbreviations: ASR, standardized incidence rate; CIR, crude incidence rate.

**FIGURE 5:**
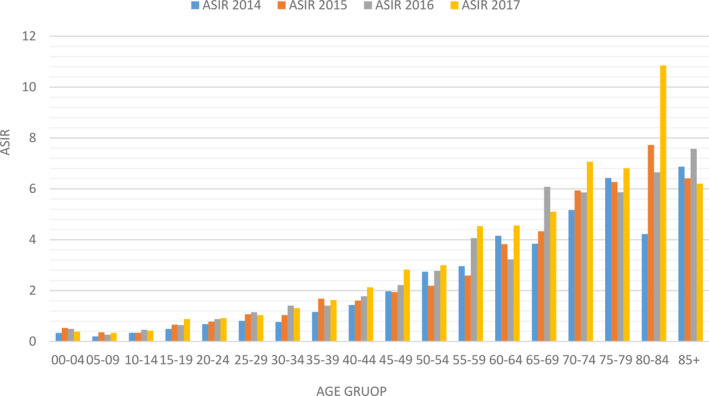
Age‐standardized incidence rate (ASIR)/100 000 of STS, 2004–2017.

The research findings reveal notable age‐specific cancer incidence rate (ASIR) shifts between 2014 and 2017. It is noteworthy that the ASIR demonstrates a consistent pattern with age, with the most pronounced gains observed in the 55–59 age group, reaching a peak of 4.535 in 2017. Furthermore, individuals aged 80–84 exhibited a pronounced elevation in ASIR, reaching a peak of 10.848 in the same year. This indicates a direct correlation between elevated cancer risk and advancing age (Figure 5 and Tables S1–S4).

## 6 Discussion

In this study, a total of 4968 cases of STS registered in the Cancer Registry System between 2014 and 2017 were examined. The research yielded the following standardized incidence rates of STS: 1.25, 1.36, 1.37, and 1.78, respectively, for the period 2014–2017. The normalized incidence rate of STS in Arbid, northern Jordan, was estimated by Alorjani et al.'s study [12] in Jordan in 2022 to be 1.37 per 100 000 inhabitants. This is somewhat less than the worldwide range, which varied from 1.4–5 per 100 000 individuals, recorded in some previous research [13]. A recent study conducted in China revealed that STSs lacking GIST20 exhibited a standardized incidence rate of 1.85 per 100 000 figure that exceeds our ASR. The overall ASR for BS in Italy was 7.0 per 100 000 from 2009 to 2012, whereas the ASR for STS was estimated to be 1.7 per 100 000. With regard to geographical distribution, the highest ASR (2.4) was observed in central Italy, while the lowest (1.6) was noted in southern Italy. The age‐standardized rate (ASR) for BS was highest in central Italy (1.1), while northern Italy had the lowest rate (7.0). In 2013, the European estimates for ASR indicated an average incidence rate of 4 per 100 000 for adult STS and sarcomas of the digestive organs (excluding GIST) [14].

The limited occurrence of STS and the use of diverse criteria and categorization systems for STS across countries and periods may contribute to the disparity in STS rates across different countries. We provide more competitive prices than those in Europe. In the United States, the incidence rate reported by Toro et al. was 5 per 100 000 in 2006 and has lately decreased to 3.4 per 100 000 [15]. The Netherlands had a high incidence rate of STS at 4.7 per 100 000 between 1950 and 1988 [16]. The high incidence rates of STS in the United States and the Netherlands are among the highest worldwide, perhaps due to the high occurrence of Kaposi's sarcoma in both countries. A population‐based research in Karachi, Pakistan, found an ASIR of 3.3 per 100 000 for men and 2.1 per 100 000 for females from 1995 to 1997. Karachi has high rates of STS, which categorizes it as a high‐risk location. The rates are similar to those in the United States and Europe and higher than in the rest of the South Asian population [17]. Their ASR values exceed ours, at a rate of 1.47 per 100 000 for males. The study conducted in Serbia's Vojvodina area found that the average ASIR of STSs was 1.9 per 100 000 between 1985 and 2009.

There was a gradual rise in rates throughout the years, ranging from 2.4 per 100 000 in 1990–2.7 per 100 000 in 1997. The average yearly percentage change throughout this time was 0.77% [18]. In Austria, the incidence rate of STS is reported to be 2.4 per 100 000 [19], more significant than the rate in this research. The current research shows a rising incidence rate during the study period, mirroring results from the Netherlands and Serbia. The age‐standardized incidence rate (ASIR) for STS in males from 2014 to 2017 was 1.47, 1.46, 1.52, and 1.98, respectively. The incidence rates for women were 1.06, 1.29, 1.21, and 1.58 throughout the same period. The data clearly demonstrate that STS are more prevalent in males than in females. Salarie et al. conducted a research project on the epidemiology of STS in Yazd between 1994 and 2005, reporting a total of 93 cases. Of the total number of patients included in the study, 58.1% were male, indicating a greater involvement of men in the research sample. [20] From 2008 to 2019, Ebrahimpour and colleagues in Iran conducted research into STS of the extremities. The study included a total of 2593 individuals, comprising 1476 males and 1117 females. The ASIR for overall STS was calculated to be 6.34 per million per year. The male predominance was found to be statistically significant (*p* < 0.05) in STS of the extremities, which is consistent with the findings of our research [14].

A retrospective analysis of 498 primary STS cases at Cukurova University, Turkey (1999–2010) revealed that leiomyosarcoma was the most common subtype (23%). Most cases were located in the lower extremity (24.7%) and uterine region (12.4%). Only 13.1% of patients presented early, while 10.2% had locally advanced disease. Treatment included neoadjuvant/adjuvant chemotherapy (12%) and palliative chemotherapy (7.2%), with the MAID regimen being the most common (17.6%). Overall survival was 45 months, with women surviving longer (55 months) than men (36 months). Radical surgery showed better outcomes (37 months) than conservative surgery (22 months). Patients with locally advanced STS had the highest survival rates (72 months), highlighting the importance of multimodal treatment strategies, particularly radical surgery [21].

Similarly, over a six‐year period, 1477 pathologically confirmed chest wall sarcomas were recorded in the research conducted by Rahmani Seraji et al. to examine chest wall sarcomas and their epidemiological features in Iran from 2008 to 2014. A total of 581 female and 896 male cases were identified. Of all the provinces, Khuzestan exhibits the most significant prevalence of chest wall sarcomas. Furthermore, the ASIR was 1.94 per 100 000, with a higher incidence and the most significant frequency observed in males over the age of 65. [22] These results corroborate our findings, which indicate that males are more prone to developing STS than females. Moreover, the research conducted by Moghimi and colleagues over a ten‐year period from 2006 to 2016 revealed that head and neck sarcomas exhibited the highest male‐to‐female ratio, at 1.4. [23]

The discrepancy in the spatial distribution of the ASIR of sarcomas between the 2014–2015 and 2016–2017 periods may be attributed to a multitude of variables. These factors encompass changes in population density, demographic trends, medical technology advancements, diagnostic criteria modifications, increased awareness of sarcomas among healthcare providers, natural temporal fluctuations in disease incidence, variations in healthcare access and reporting practices, environmental influences, and random variation in disease occurrence. The occurrence of sarcomas exhibits geographic and temporal fluctuations, and the distribution of histological subgroups remains poorly defined. Furthermore, the observed clustering patterns may be influenced by over 150 distinct histological subtypes of sarcomas. It is possible that the national registry and the precision of data‐gathering techniques may impact the identified clustering pattern variations. Therefore, the change in the regional distribution of sarcoma cases between the two time periods is likely influenced by a combination of these factors [8].

The analysis demonstrated a persistent increase in ASIR with advancing age, with the most notable elevations observed in the 55–59 age cohort, reaching a peak of 4.535 in 2017, and the 80–84 age group, which reached a maximum of 10.848 in the same year. The incidence of STS increases with age, with the annual rate rising from 0.9 per 100 000 in children under 10–18.2 per 100 000 in adults over 60 years of age [24]. The research found that STS per million occurrence rates were more significant in those over 65 than in other age groups [14]. An analysis of the Surveillance Epidemiology and End Results database revealed that persons under 20 accounted for 5.6% of STS cases, with rhabdomyosarcoma being the predominant subtype [24]. The results are consistent with the hypothesis that STS predominantly affects the elderly, with the highest prevalence observed in the senior population. The increase in the incidence of STS with advancing age is attributable to a number of factors, including cumulative exposure to carcinogenic substances, genetic alterations, and alterations in the body's microenvironment over time. Age‐related alterations in the immune system and DNA repair mechanisms may contribute to an increased prevalence of STS in the elderly. Nevertheless, the precise causes for the rise in STS incidence with age may differ depending on the subtype and require further investigation for a comprehensive understanding.

In the Karbala study, Iraq (2012–2020), 2.97% of the 7468 registered cancer cases were STS, a figure comparable to our findings. However, the overall incidence rate of STS in Karbala was slightly lower. Consistent with global trends, more common cancers like breast cancer (24.49%) and lymphomas (8.90%) had much higher incidence rates. Our study and the Karbala study found an increased incidence of STS with age, especially in those over 55. A male predominance was observed in our research, and the Karbala study similarly reported a higher incidence in males, reflecting a similar gender pattern in STS prevalence [25].

It is important to bear in mind a number of limitations associated with this research when considering the conclusions that can be drawn from it. The veracity of the findings from the Iranian National Cancer Registry is contingent upon the quality and completeness of the data, which may be susceptible to potential errors, misclassification, or underreporting. The research acknowledges the existence of numerous histological subgroups within the category of STS. However, the lack of detailed studies focusing on specific subtypes limits the depth of discoveries that can be made. The temporal resolution of the research, which spans 4 years, may not be sufficient to capture short‐term fluctuations in the incidence of STS. A longer study period may facilitate a more comprehensive understanding of temporal variability. The absence of investigation into potential risk factors associated with STS constrains the ability to ascertain the etiology of the disease. The research does not provide detailed information on the underlying causes of the clustering patterns identified by the geographical analysis utilizing Moran's Index. These may include socioeconomic variables, healthcare access, or environmental factors.

## 7 Conclusion

The incidence of STS in Iran is lower than the global average. A slight increase in the incidence of STS was observed during the research, which is in line with global trends. There is a greater likelihood of males experiencing this condition than females. Moreover, when comparing cities with different geographical and climatic characteristics, these factors may influence the development of this malignant disease. The discrepancies in gender disparity, regional distribution, and incidence rates underscore the complexity of STS. The findings of this study may assist healthcare professionals and policymakers in developing region‐specific plans for the treatment, early detection, and prevention of STS. To enhance our understanding of this rare form of cancer within the Iranian population, it is imperative to conduct ongoing research and monitoring to elucidate the underlying mechanisms that contribute to the observed trends. It is recommended that pertinent information on recurrent risk factors be gathered and assessments be conducted on a broader scale than just the epidemiology of STS in collaboration with cancer centers throughout the Middle East. This will facilitate improving the evaluation of the disease's burden and trends.

## Author Contributions

M.D.B. and N.H. planned and coordinated the research. V.R. and S.H. conducted the literature search and screening. N.H. and S.H. collected the data, and S.H. performed the statistical analysis. V.R., N.H., and S.H. all contributed to the interpretation of the data. V.R. and S.H. wrote the manuscript's first draft, while N.H. made substantial changes afterward. All authors have reviewed and approved the final paper. N.H. had complete access to the research data and assumed full responsibility for guaranteeing its quality and integrity.

## Ethics Statement

It states that the research was submitted to the Shahid Sadoughi University of Medical Sciences Ethics Committee and was authorized under the number IR.SSU.MEDICINE.REC.1400.028. The study's data came from the cancer registry system using unique identifiers.

## Conflicts of Interest

The authors declare no conflicts of interest.

## Supporting information


Data S1.

Data S2.

## Data Availability

Upon reasonable request, the corresponding author may provide access to the data supporting the study's results. The data used in this investigation are accessible upon request. The corresponding author may be reached by Email at [Narjeshazar@yahoo.com] for researchers interested in gaining access to the data. We are committed to following the ethical and legal data‐sharing guidelines while making our data available for scholarly and research purposes.
