# Supplementary material for: Epidemiology of Soft Tissue Sarcoma in Iran: Four‐Year National Cancer Registry Data Report (2014–2017)
Source: Cancer Rep (Hoboken). 2025 Jan 10;8(1):e70118. doi: 10.1002/cnr2.70118 (PMC11726690; doi:10.1002/cnr2.70118)
Supplement: Supplementary file 1 — Data S1. [file CNR2-8-e70118-s001.docx]

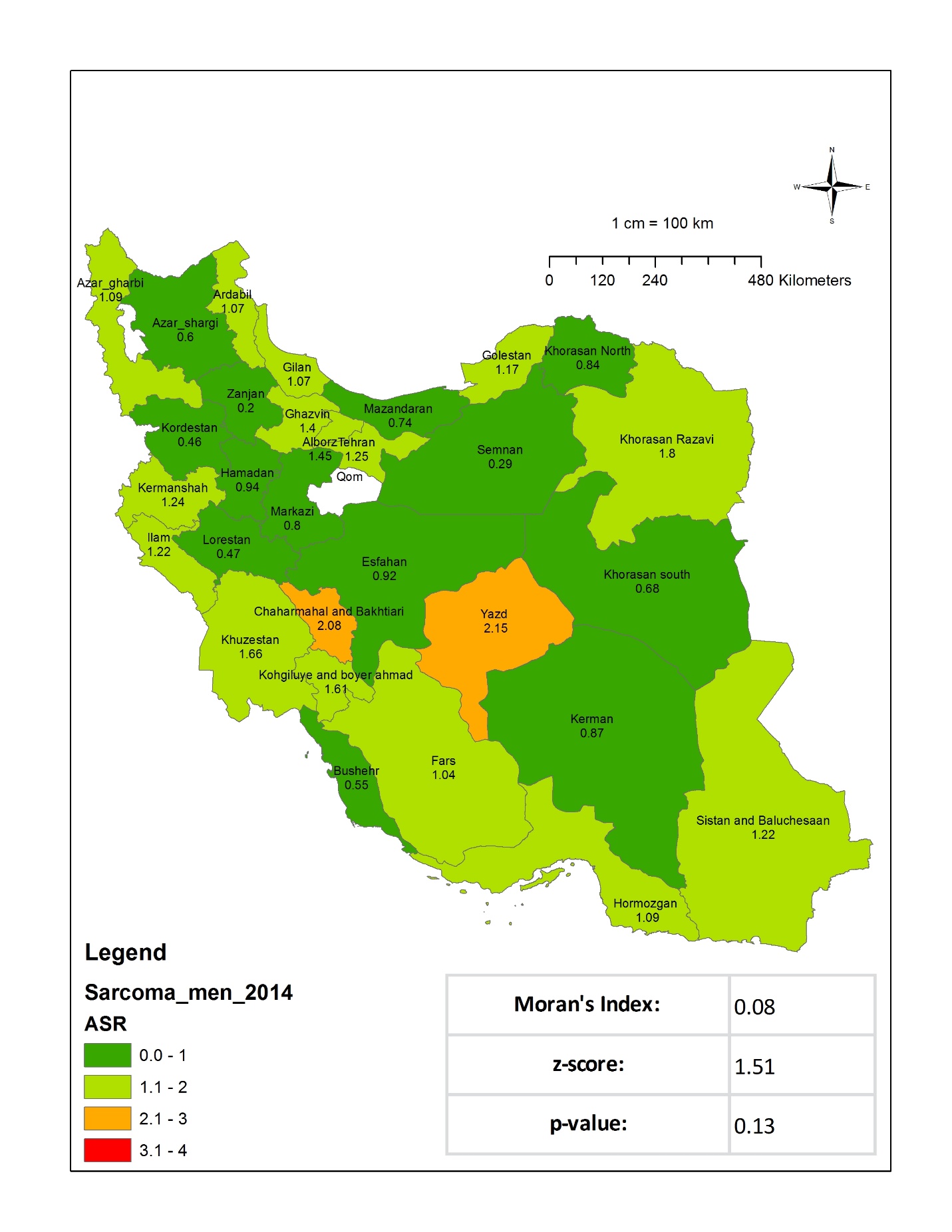


Supplementary Fig 1: Age-Standardized Incidence Rate (ASR) of Soft Tissue Sarcoma per 100,000 populations for male in Iran in 2014


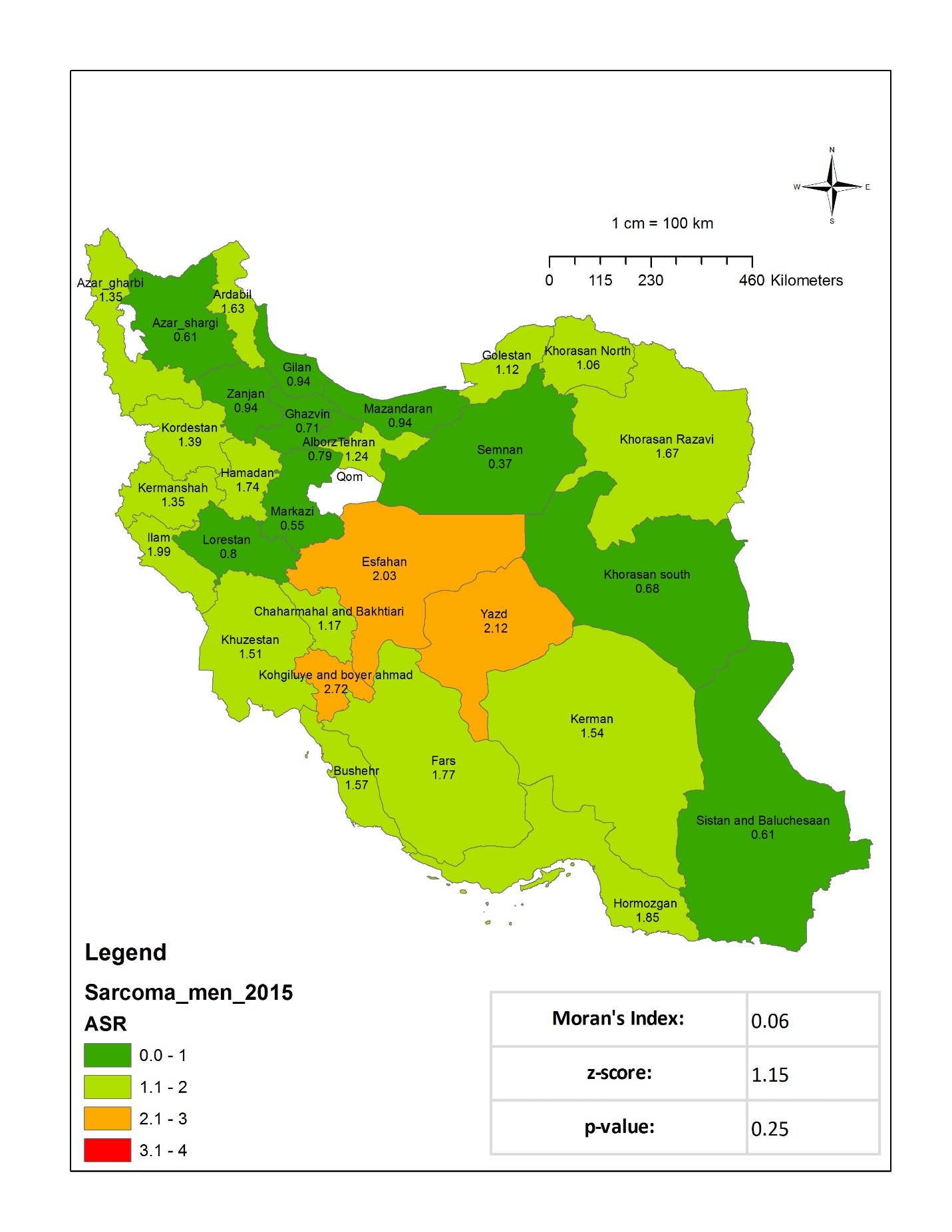


Supplementary Fig 2: Age-Standardized Incidence Rate (ASR) of Soft Tissue Sarcoma per 100,000 populations for male in Iran in 2015


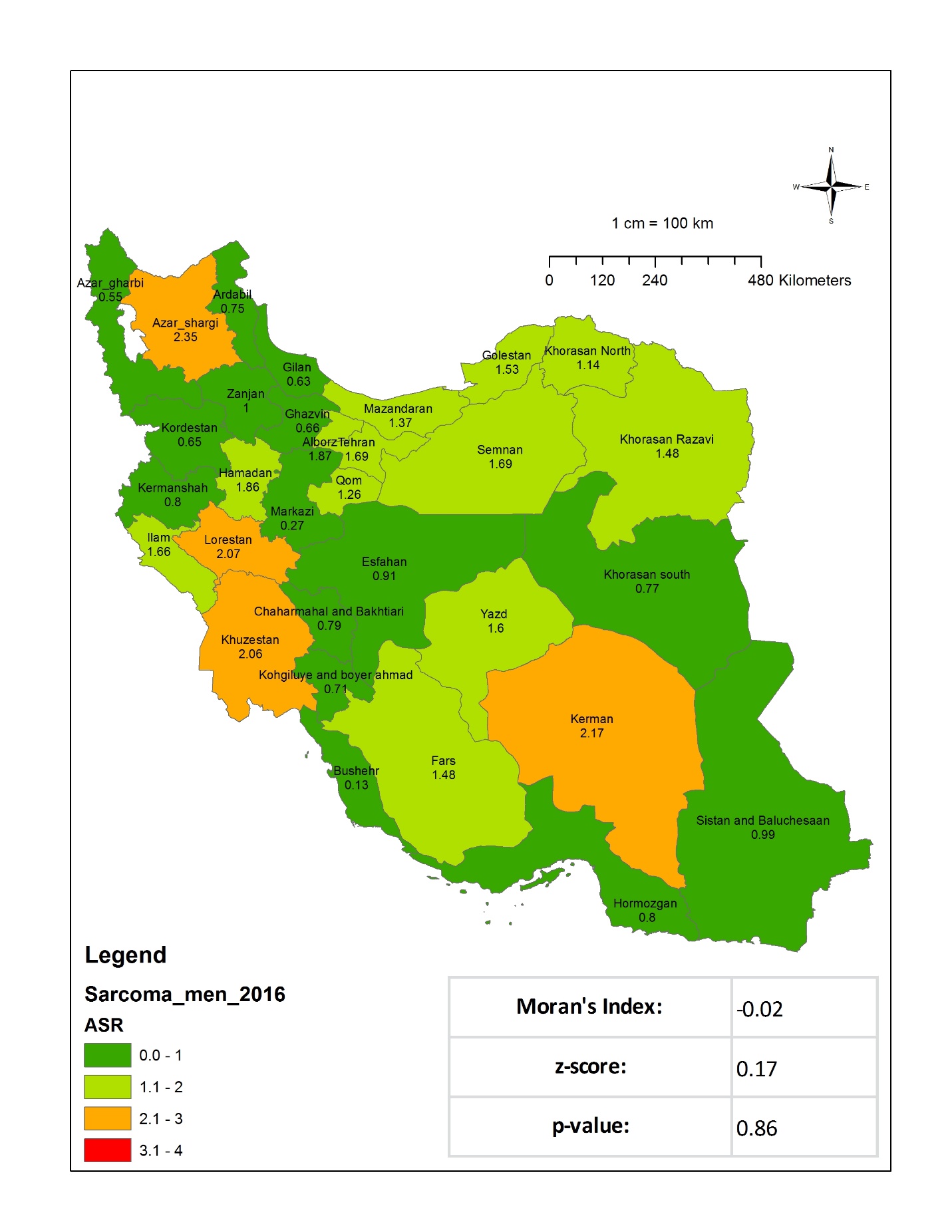


Supplementary Fig 3: Age-Standardized Incidence Rate (ASR) of Soft Tissue Sarcoma per 100,000 populations for male in Iran in 2016


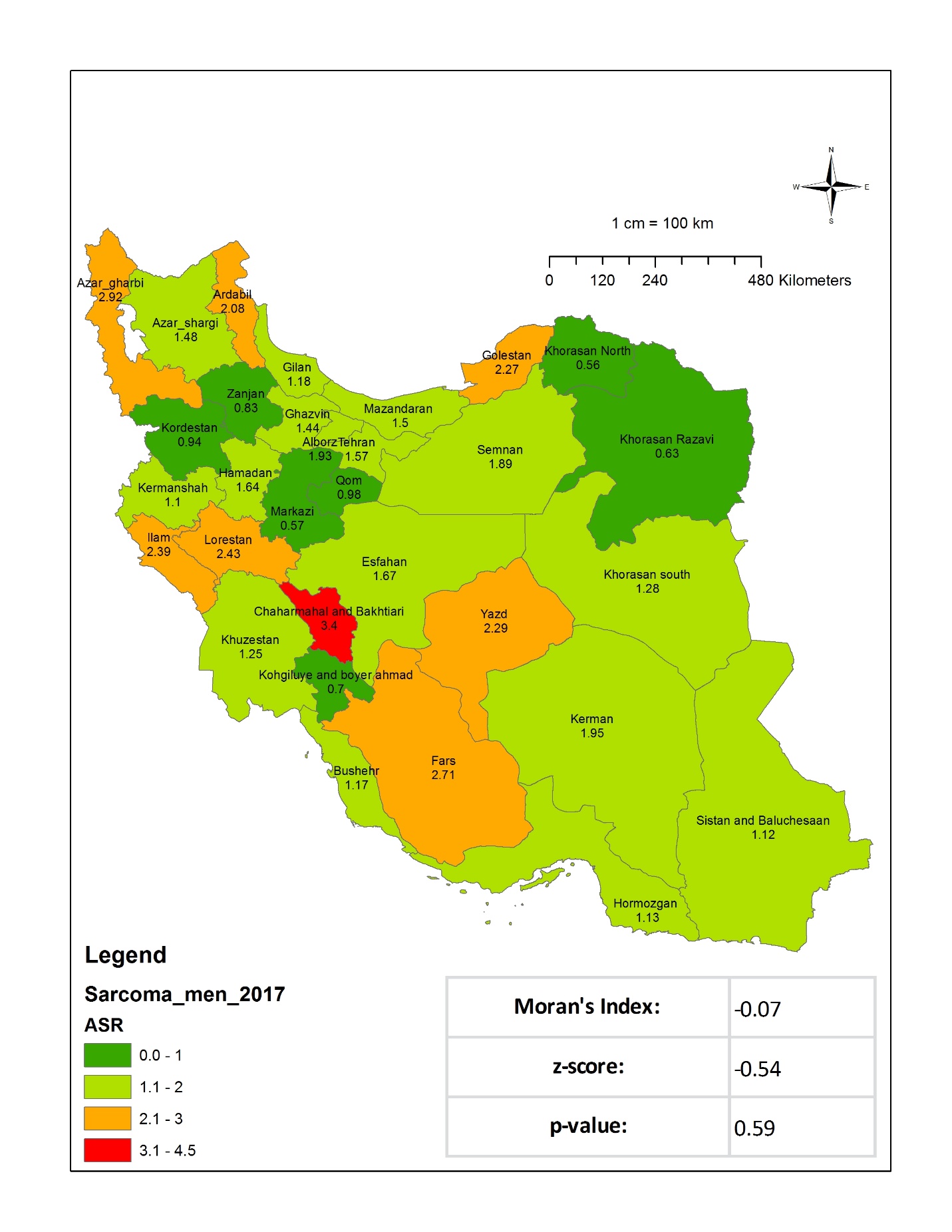


Supplementary Fig 4: Age-Standardized Incidence Rate (ASR) of Soft Tissue Sarcoma per 100,000 populations for male in Iran in 2017


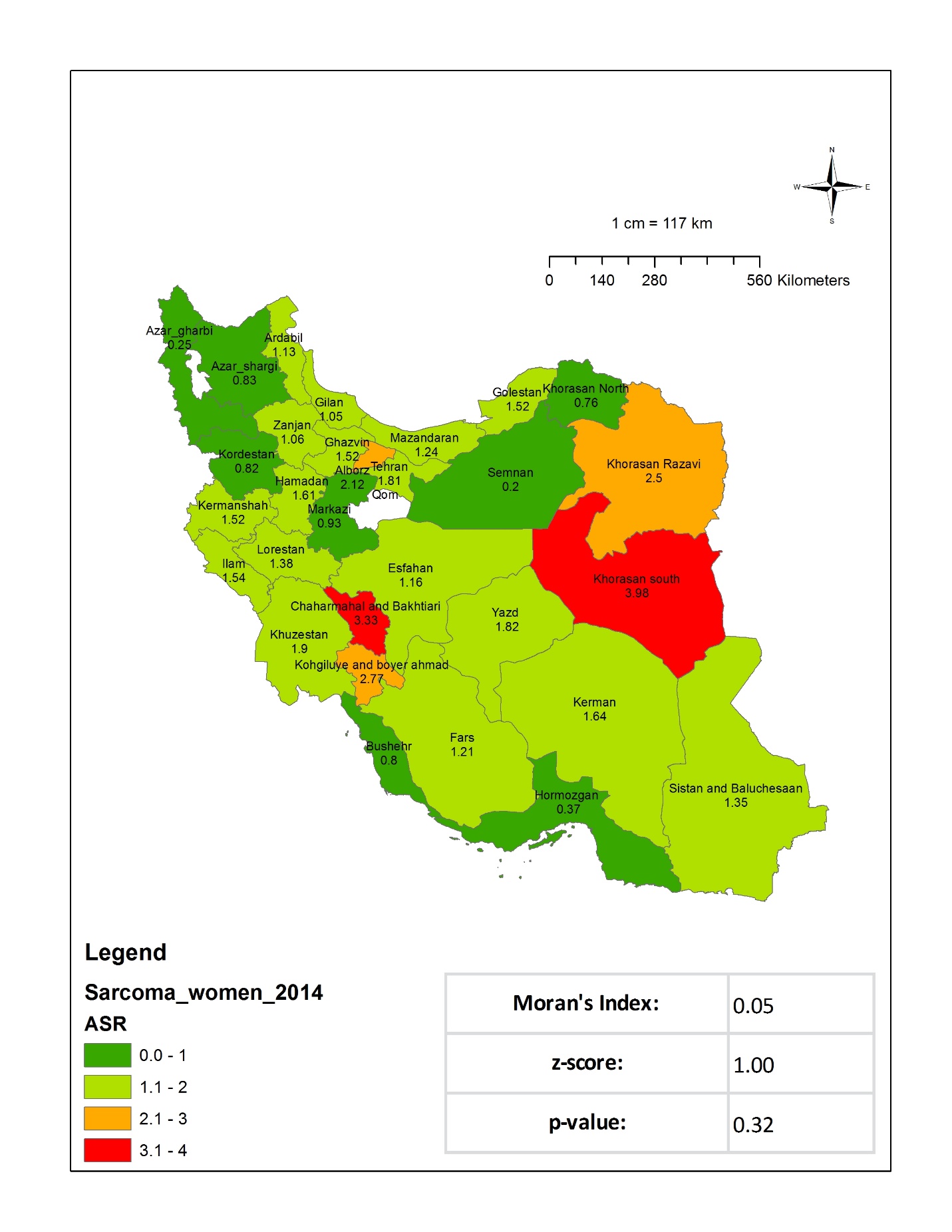


Supplementary Fig 5: Age-Standardized Incidence Rate (ASR) of Soft Tissue Sarcoma per 100,000 populations for female in Iran in 2014


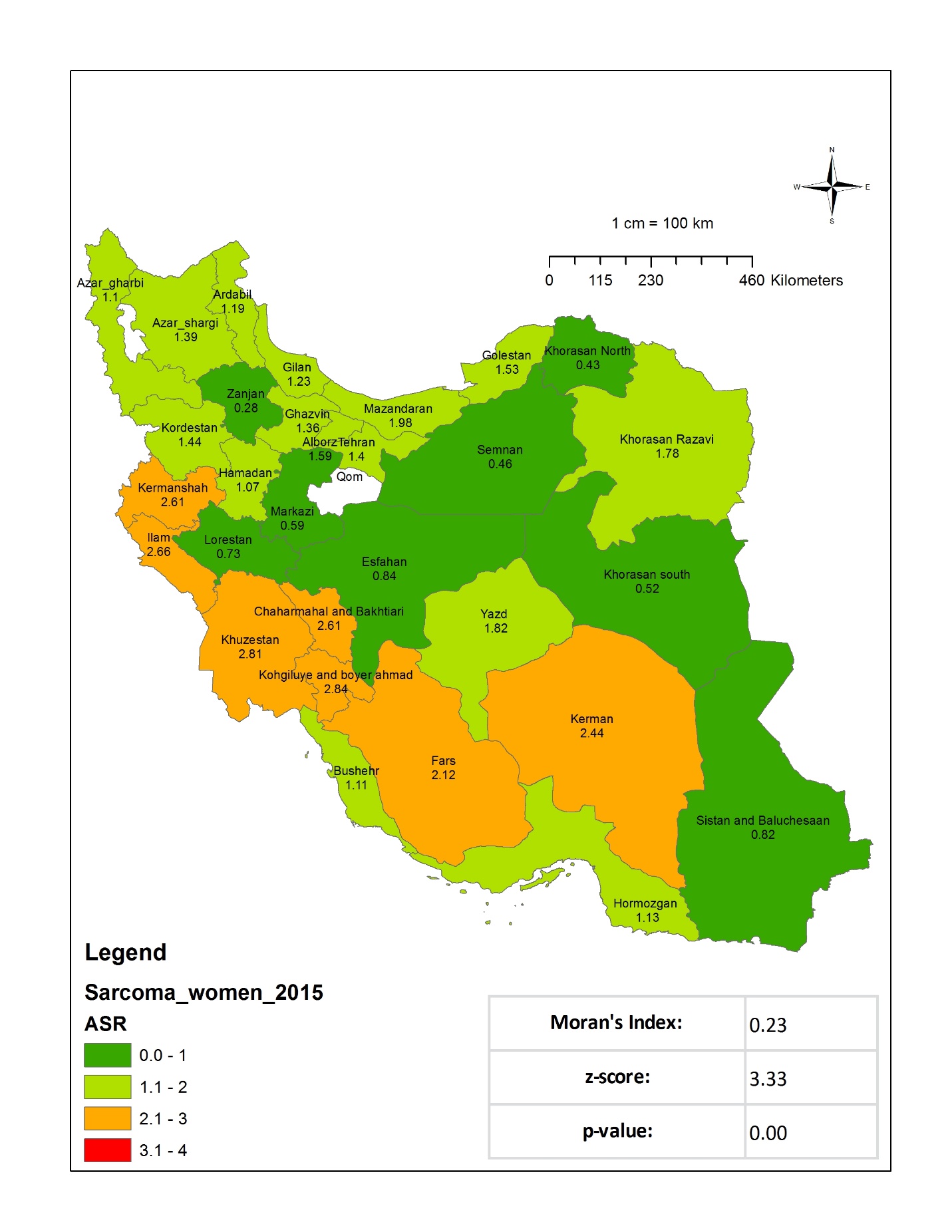


Supplementary Fig 6: Age-Standardized Incidence Rate (ASR) of Soft Tissue Sarcoma per 100,000 populations for female in Iran in 2015


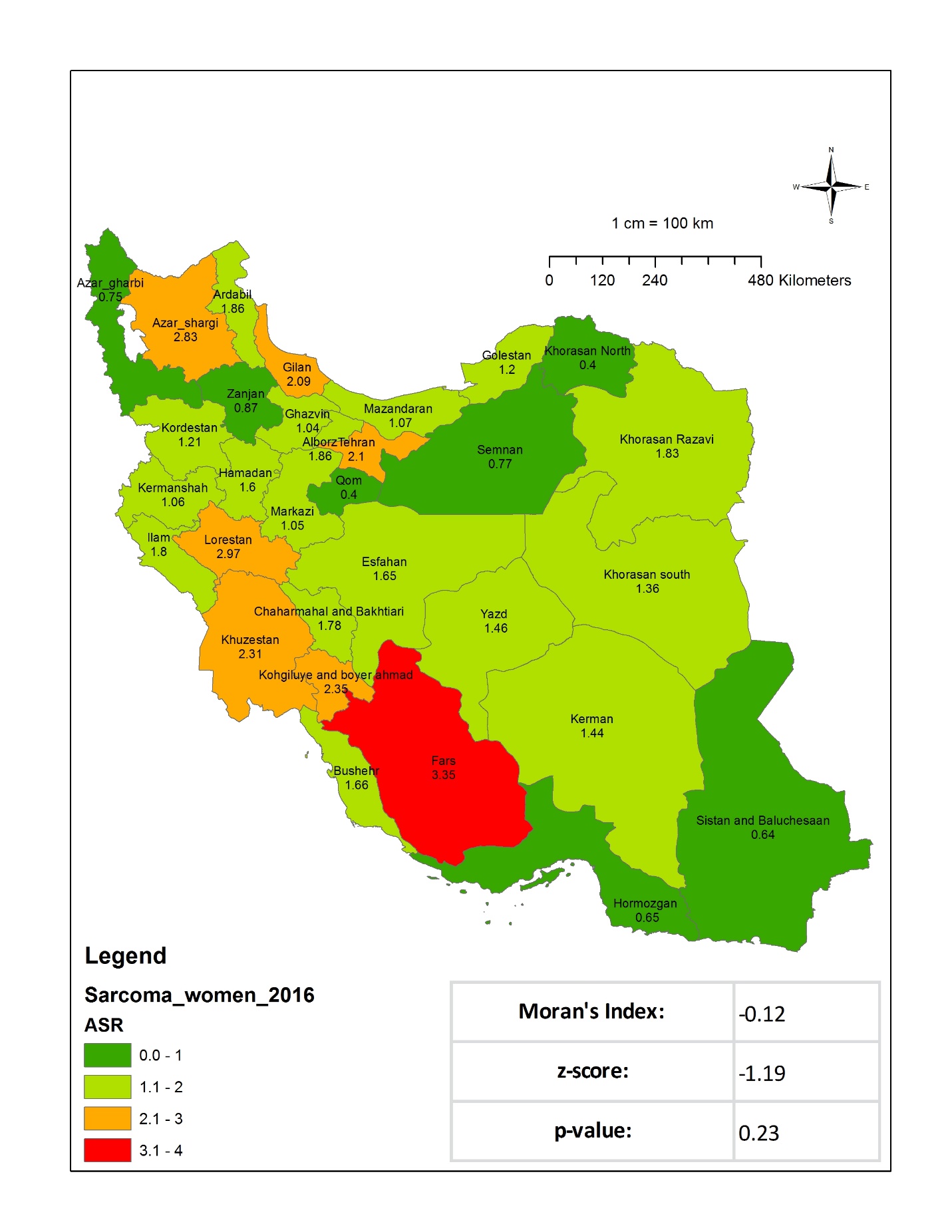


Supplementary Fig 7: Age-Standardized Incidence Rate (ASR) of Soft Tissue Sarcoma per 100,000 populations for female in Iran in 2016


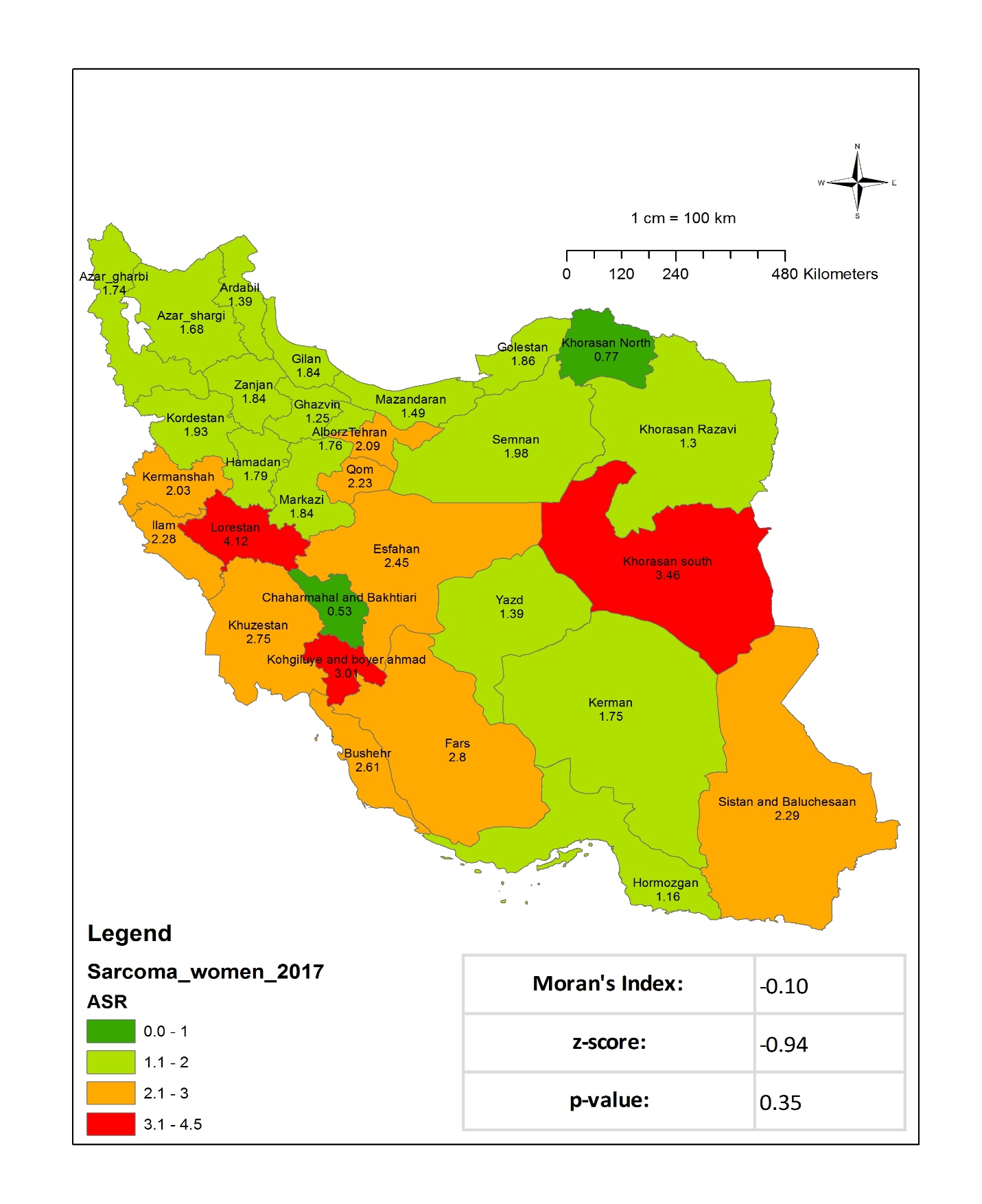


Supplementary Fig 8: Age-Standardized Incidence Rate (ASR) of Soft Tissue Sarcoma per 100,000 populations for female in Iran in 2017
