# Supplementary material for: Epidemiology of Soft Tissue Sarcoma in Iran: Four‐Year National Cancer Registry Data Report (2014–2017)
Source: Cancer Rep (Hoboken). 2025 Jan 10;8(1):e70118. doi: 10.1002/cnr2.70118 (PMC11726690; doi:10.1002/cnr2.70118)
Supplement: Supplementary file 2 — Data S2. [file CNR2-8-e70118-s002.docx]

**Supplementary table1:** Age-Specific Incidence Rate (ASIR) for STS in 2014

| **Age, years** | **Count** | **Population** | **Age-Specific Incidence Rate** |
| --- | --- | --- | --- |
| 00-04 | 22 | 6620000 | 0.332326284 |
| 05-09 | 12 | 6030000 | 0.199004975 |
| 10-14 | 19 | 5568000 | 0.341235632 |
| 15-19 | 29 | 5903000 | 0.491275623 |
| 20-24 | 50 | 7323000 | 0.682780281 |
| 25-29 | 71 | 8748000 | 0.811614083 |
| 30-34 | 62 | 8095000 | 0.76590488 |
| 35-39 | 73 | 6290000 | 1.160572337 |
| 40-44 | 75 | 5235000 | 1.432664756 |
| 45-49 | 89 | 4518000 | 1.969898185 |
| 50-54 | 103 | 3760000 | 2.739361702 |
| 55-59 | 93 | 3145000 | 2.957074722 |
| 60-64 | 93 | 2239000 | 4.153640018 |
| 65-69 | 58 | 1510000 | 3.841059603 |
| 70-74 | 57 | 1103000 | 5.167724388 |
| 75-79 | 55 | 856000 | 6.425233645 |
| 80-84 | 22 | 521000 | 4.222648752 |
| 85+ | 27 | 393000 | 6.870229008 |
| All Ages | 1010 | 77856000 | 1.297266749 |

**Supplementary table2:** Age-Specific Incidence Rate (ASIR) for STS in 2015

| **Age, years** | **Count** | **Population** | **Age-Specific Incidence Rate** |
| --- | --- | --- | --- |
| 00-04 | 36 | 6760000 | 0.532544379 |
| 05-09 | 22 | 6129000 | 0.358949258 |
| 10-14 | 19 | 5596000 | 0.339528234 |
| 15-19 | 38 | 5763000 | 0.659378796 |
| 20-24 | 54 | 6925000 | 0.779783394 |
| 25-29 | 92 | 8593000 | 1.070638892 |
| 30-34 | 87 | 8406000 | 1.034975018 |
| 35-39 | 111 | 6595000 | 1.683093252 |
| 40-44 | 86 | 5358000 | 1.605076521 |
| 45-49 | 91 | 4690000 | 1.940298507 |
| 50-54 | 84 | 3837000 | 2.189210321 |
| 55-59 | 85 | 3282000 | 2.589884217 |
| 60-64 | 91 | 2380000 | 3.823529412 |
| 65-69 | 69 | 1593000 | 4.331450094 |
| 70-74 | 66 | 1112000 | 5.935251799 |
| 75-79 | 53 | 846000 | 6.264775414 |
| 80-84 | 40 | 518000 | 7.722007722 |
| 85+ | 25 | 390000 | 6.41025641 |

**Supplementary table3:** Age-Specific Incidence Rate(ASIR) for STS in 2016

| **Age, years** | **Count** | **Population** | **Age-Specific Incidence Rate** |
| --- | --- | --- | --- |
| 00-04 | 35 | 7093004 | 0.493443963 |
| 05-09 | 17 | 6411277 | 0.265157784 |
| 10-14 | 26 | 5688384 | 0.457071815 |
| 15-19 | 35 | 5458997 | 0.641143419 |
| 20-24 | 56 | 6392879 | 0.875974659 |
| 25-29 | 94 | 8201133 | 1.146183094 |
| 30-34 | 121 | 8600913 | 1.406827391 |
| 35-39 | 99 | 7037598 | 1.406729967 |
| 40-44 | 98 | 5518307 | 1.775906995 |
| 45-49 | 107 | 4833123 | 2.213889446 |
| 50-54 | 109 | 3925971 | 2.776383218 |
| 55-59 | 136 | 3350593 | 4.058982992 |
| 60-64 | 82 | 2542573 | 3.225079477 |
| 65-69 | 104 | 1711464 | 6.076668864 |
| 70-74 | 69 | 1177625 | 5.85925061 |
| 75-79 | 52 | 886392 | 5.866478939 |
| 80-84 | 43 | 646733 | 6.64880252 |
| 85+ | 34 | 449304 | 7.567259584 |

**Supplementary table 4**: Age-Specific Incidence Rate(ASIR) for STS in 2017

| **Age, years** | **Count** | **Population** | **Age-Specific Incidence Rate** |
| --- | --- | --- | --- |
| 00-04 | 28 | 7264370 | 0.385442922 |
| 05-09 | 22 | 6561310 | 0.335298896 |
| 10-14 | 24 | 5691105 | 0.421710722 |
| 15-19 | 46 | 5228623 | 0.879772743 |
| 20-24 | 55 | 5987536 | 0.918574853 |
| 25-29 | 84 | 8105731 | 1.03630382 |
| 30-34 | 117 | 8925826 | 1.310803056 |
| 35-39 | 119 | 7330218 | 1.62341693 |
| 40-44 | 120 | 5639991 | 2.12766297 |
| 45-49 | 141 | 4993124 | 2.823883404 |
| 50-54 | 120 | 4005227 | 2.996084866 |
| 55-59 | 158 | 3484337 | 4.534578601 |
| 60-64 | 122 | 2678271 | 4.555177575 |
| 65-69 | 91 | 1784834 | 5.098513363 |
| 70-74 | 84 | 1189012 | 7.064689002 |
| 75-79 | 60 | 880854 | 6.811571498 |
| 80-84 | 72 | 663703 | 10.84822579 |
| 85+ | 29 | 467720 | 6.200290772 |
